# Supplementary material for: Insulin Resistance Promotes Parkinson’s Disease through Aberrant Expression of α-Synuclein, Mitochondrial Dysfunction, and Deregulation of the Polo-Like Kinase 2 Signaling
Source: Cells. 2020 Mar 17;9(3):740. doi: 10.3390/cells9030740 (PMC7140619; doi:10.3390/cells9030740)
Supplement: Supplementary file 1 [file cells-09-00740-s001.pdf]

cells

MDPI

Article

Insulin Resistance Promotes Parkinson's Disease through Aberrant Expression of α-Synuclein, Mitochondrial Dysfunction and Deregulation of the Polo-Like Kinase 2 Signaling

Supplementary Materials

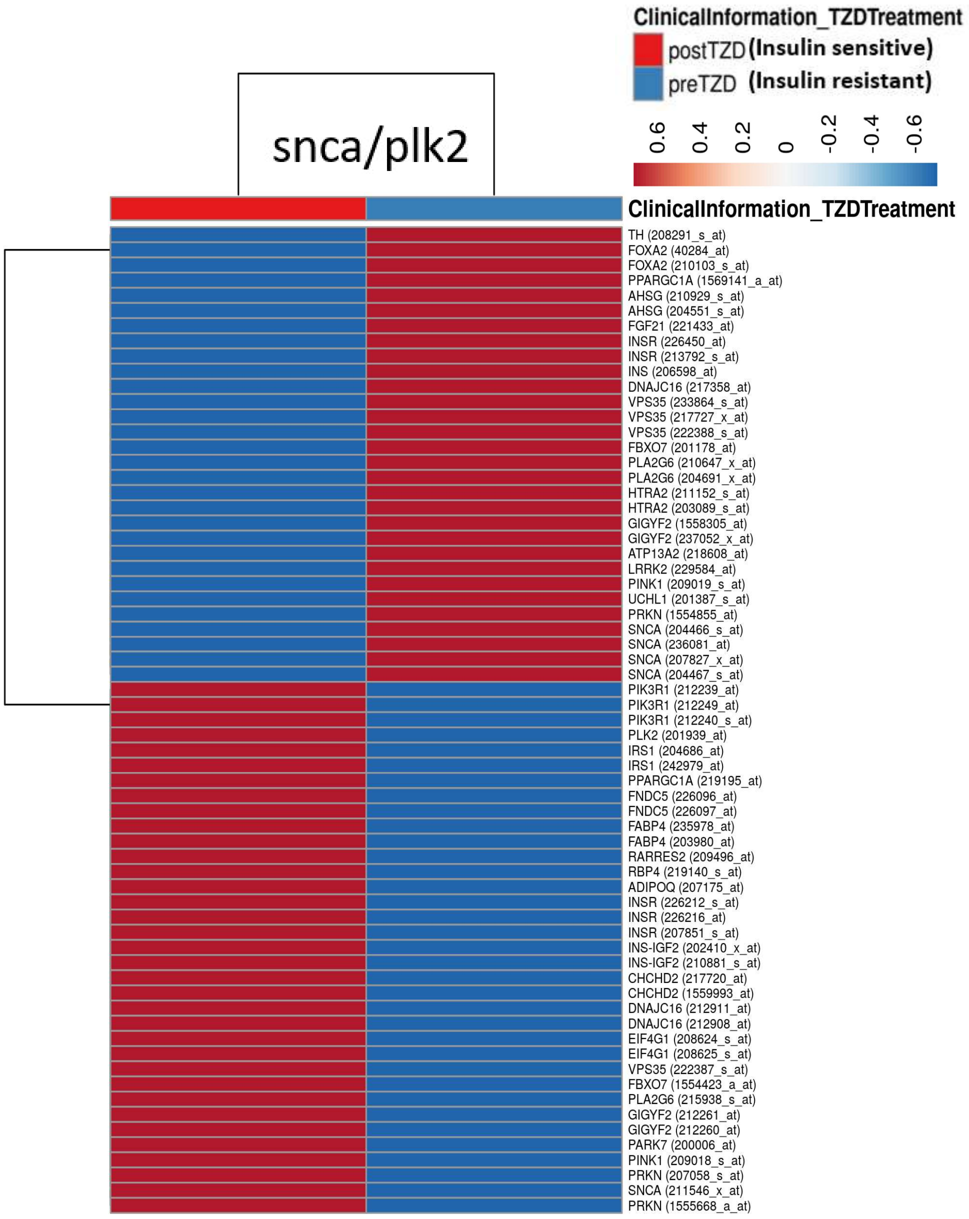

Figure S1. Patients' genomic landscape indicates a nosological association between Diabetes and PD, in silico. PD and IR-related gene expression heatmap from reanalysis of human insulin resistance and thiazolidinedone-mediated insulin sensitization dataset (Homo sapiens, A-AFFY-44, AFFY_HG_U133_PLUS_2, E-GEOD-13070, 11 samples, 54675 genes). Rows are centered; unit variance scaling is applied to rows. Both rows and columns are clustered using correlation distance and average linkage. 65 rows, 11 columns.

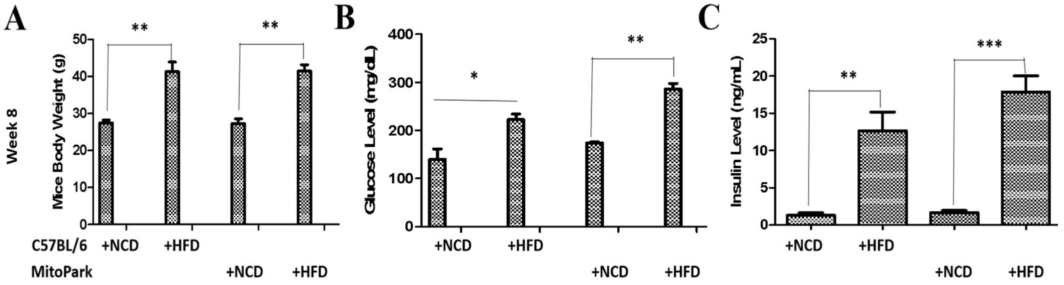

Figure S2. Indicators of insulin resistance. Graphical representation of the week 8 differential (**A**) body weight, (**B**) plasma glucose, and (**C**) insulin level of C57BL/6 or MitoPark mice fed on normal chow diet or high fat diet for 8 weeks. ns, $p > 0.05$, *, $p < 0.05$, **, $p < 0.01$, ***, $p < 0.001$. NCD, normal chow diet; HFD, high fat diet.

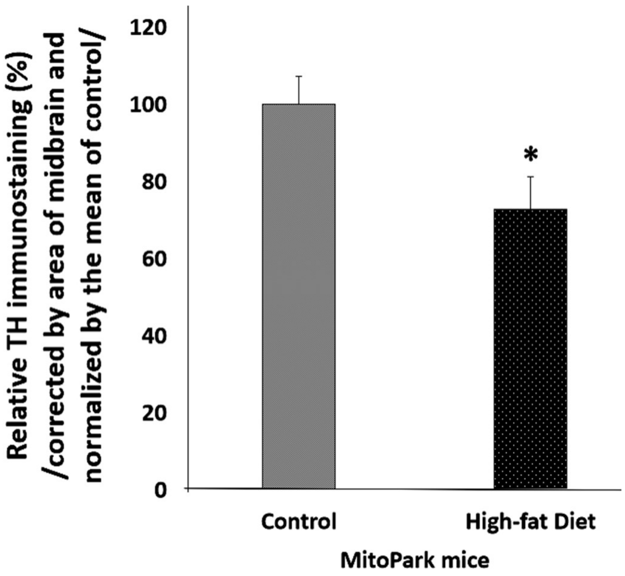

Figure S3. Graphical representation of the differential TH immunostaining in midbrain samples from NCD-fed control and HFD Mitopark mice. *, $p < 0.05$; NCD, normal chow diet; HFD, high fat diet.

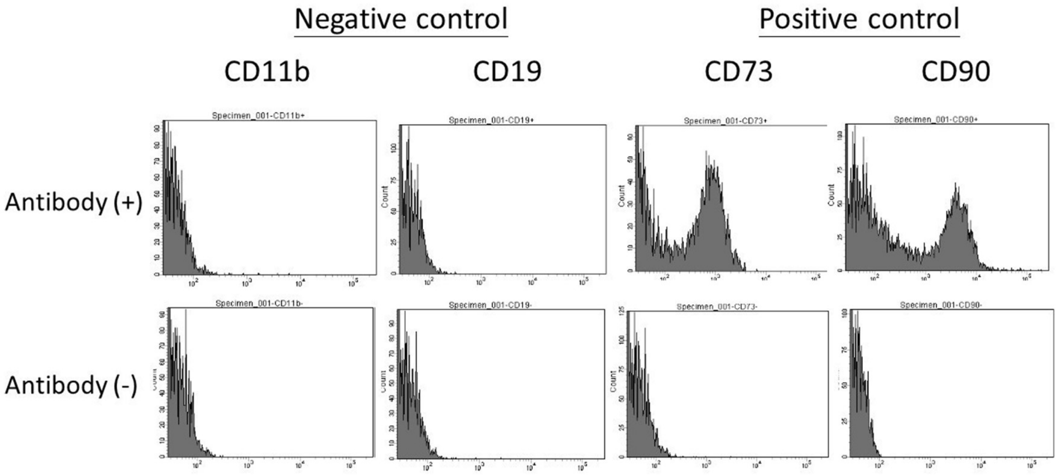

Figure S4. Surface marker-based characterization of adipocyte-derived stem cells (ADSCs). Flow cytometry isolation of ADSCs based on CD73/CD90 surface marker immunopositivity and CD11b/CD19 negativity. The stained isolated ADSCs were compared with non-stained control under the same fluorescent intensity to avoid the auto-fluorescence.
